# **Postprandial leucine and insulin responses and toxicological effects of a novel whey protein hydrolysate-based supplement in rats**

**DOI:** 10.1186/1550-2783-9-24

**Published:** 2012-06-06

**Authors:** Ryan G Toedebusch, Thomas E Childs, Shari R Hamilton, Jan R Crowley, Frank W Booth, Michael D Roberts

**Affiliations:** 1 Department of Biomedical Sciences, College of Veterinary Medicine, University of Missouri, Columbia, MO, USA; 2 Research Animal Diagnostics Laboratory (RADIL), Columbia, MO, USA; 3 NIH/NCRR Mass Spectrometry Resource, Washington University, St. Louis, MO, USA; 4 School of Medicine, Division of Endocrinology, Metabolism, Lipid Research, Washington University, St. Louis, MO, USA; 5 Dalton Cardiovascular Research Center, University of Missouri, Columbia, MO, USA; 6 Department of Medical Pharmacology and Physiology, School of Medicine, University of Missouri, Columbia, MO, USA; 7 Department of Nutrition and Exercise Physiology, College of Environmental Health Sciences, University of Missouri, Columbia, MO, USA

**Keywords:** Diet, Leucine, Whey hydrolysate

## Abstract

The purpose of this study was: aim 1) compare insulin and leucine serum responses after feeding a novel hydrolyzed whey protein (WPH)-based supplement versus a whey protein isolate (WPI) in rats during the post-absorptive state, and aim 2) to perform a thorough toxicological analysis on rats that consume different doses of the novel WPH-based supplement over a 30-day period. In male Wistar rats (~250 g, n = 40), serum insulin and leucine concentrations were quantified up to 120 min after one human equivalent dose of a WPI or the WPH-based supplement. In a second cohort of rats (~250 g, n = 20), we examined serum/blood and liver/kidney histopathological markers after 30 days of feeding low (1human equivalent dose), medium (3 doses) and high (6 doses) amounts of the WPH-based supplement. In aim 1, higher leucine levels existed at 15 min after WPH vs. WPI ingestion (p = 0.04) followed by higher insulin concentrations at 60 min (p = 0.002). In aim 2, liver and kidney histopathology/toxicology markers were not different 30 days after feeding with low, medium, high dose WPH-based supplementation or water only. There were no between-condition differences in body fat or lean mass or circulating clinical chemistry markers following the 30-day feeding intervention in aim 2. In comparison to WPI, acute ingestion of a novel WPH-based supplement resulted in a higher transient leucine response with a sequential increase in insulin. Furthermore, chronic ingestion of the tested whey protein hydrolysate supplement appears safe.

## **Introduction**

More than 150 million US residents consume dietary supplements and many of those are products including whey protein, creatine, and branched-chain amino acids (BCAAs) [1]. Of the numerous marketed dietary supplements, it is well known that whey protein supplementation augments resistance training adaptations [2]. Moreover, recent evidence suggests that the consumption of whey protein elicits the greatest appearance of essential amino acids and insulin and is thus the seemingly most influential known protein source capable of augmenting muscle anabolism [2-4]. Whey protein is commercially categorized by concentration or by degree of hydrolysate [5]. Whey protein concentrate (WPC) may contain 29% to 89% total protein by volume, with the remaining kcal coming from carbohydrates and lipids, whereas whey protein isolate (WPI) composition typically exceeds 90% total protein by volume [5]. WPH is enzymatically hydrolyzed in order to obtain smaller peptide fractions from its parent WPC or WPI source and is thought to undergo more rapid gastrointestinal absorption kinetics thus potentially improving amino acid bioavailability. In support of this hypothesis, data from Tang et al. [3] indicate that circulating leucine levels were greater with ingestion of WPH versus soy or casein at 30 minutes post ingestion in humans. Power et al. [6] studied the serum insulin, phenylalanine and total branched chain amino acid responses of ingesting 45 g of WPI or WPH after an overnight fast in humans. Of the measured variables, these authors reported that WPH elicited a statistically greater phenylalanine response compared to WPI [6]. Thus, there is still conflicting evidence as to whether or not WPH elicits a more favorable serum anabolic response (i.e., greater insulin and leucine values) relative to other whey protein forms. Furthermore, limited evidence to our knowledge has compared the postprandial effects that exist between a whey protein isolate relative to a hydrolyzed whey protein derived from WPI [7]. Data comparing the effects of different protein sources on serum amino acid and hormone concentrations typically examine these phenomena after overnight fasting period, which is not applicable to those who consume supplemental protein between meals. Lockwood et al. [8] studied the effects of ingesting 60 g/day of WPH versus two different whey protein concentrate supplements on body composition after 8 weeks of progressive resistance training. The authors discovered that all three protein forms similarly affected total body muscle mass, strength, anaerobic endurance and blood lipids. However, the authors did not analyze the acute feeding serum responses [8]. Therefore, while WPH may elicit transient increases in circulating leucine and insulin relative to other protein sources, data is lacking with regard to how a WPH-based supplement affects these variables in the post-absorptive state.

Clarity is also warranted with regard to whether or not weeks-to-months of whey protein supplementation yield adverse health effects (i.e., kidney and/or liver damage). Large-scale human studies have demonstrated that higher protein intakes seemingly exert no adverse effects on markers of renal or liver function [9,10]. There are, however, equivocal safety concerns brought about through the internet and media regarding the prolonged effects of consuming copious amounts of dietary protein whether it is through high protein foods or protein supplements [11]. Likewise, there is the imminent possibility that whey protein supplement users disregard and supersede the recommended dosages and combine whey with other dietary supplement ingredients. Therefore, multiple dosages of protein supplements should be thoroughly investigated for safety of consumption.

Animal models offer a variety of advantages compared to humans to study how mammals physiologically cope with nutritional interventions. Specifically, animals’ diets can be tightly regulated, multiple tissues can be dissected and analyzed, and supplement adherence can be assured. Therefore, the purpose of the current study was two-fold: aim 1) to use a rat model to compare the post-prandial insulin and leucine responses between a novel WPH-based supplement versus a WPI powder in rats that were in the post-absorptive state, and aim 2) to perform a thorough toxicological analysis on rats that were fed low, medium, and high doses of the novel WPH-based supplement over a 30-day period in order to examine the safety of chronically consuming this protein source. We hypothesized that the tested WPH-based supplement would exhibit a superior insulin response when compared to the insulin response of WPI. Likewise, we hypothesized that leucine and insulin responses to the WPH-based protein would be superior to WPI based upon previous literature suggesting that the hydrolysis process potentially increases the digestibility of WPH [7]. Finally, we hypothesized that the supplement would not elicit adverse health effects on the measured health parameters on rats following a 30-day supplementation period.

## **Materials and Methods**

### **Animals and experimental protocols**

Male Wistar rats were obtained from Charles River Laboratory weighing 175–200 g. Rats were between 45–48 days of age when received. They were allowed 7 days to acclimatize to new housing and were maintained on a 12/12-h light/dark cycle, with food (Purinalab 5008 standard chow: 27% protein, 17% fat, 56% carbohydrates) provided *ad libitum* until the experimental testing days described below. Rats were received in 2 cohorts; the first (n = 36) was used to examine circulating post-gavage insulin and leucine responses between one human equivalent dose (low dose) of WPI and the tested (low dose) WPH-based supplement and the second (n = 20) was used to study how 30 days of feeding a low dose (1.1 g/d, or 1 human equivalent dose), medium dose (3.4 g/d, 3 human eq. doses), high dose (6.8 g/d, 6 human eq. doses) of the WPH-based supplement affected toxicological variables. The ingredients for each dose are defined in the next section. The experimental protocol was approved by the Institutional Animal Care and Use Committee of The University of Missouri-Columbia.

### **Nutritional supplement information**

The WPH-based supplement (Scivation, Inc) contains the following active ingredients: Whey protein isolate (Glanbia Nutritionals, Inc), extensively hydrolyzed whey protein concentrate (32 degree of hydrolysis; average molecular weight = 1.57 Daltons; Carbery), leucine peptides (Glanbia Nutritionals, Inc), creatine monohydrate (AlzChem Trostberg GmbH), patent-pending blend of L-citrulline, L-lysine, vitamin C and folic acid (Genysis Nutrition Labs), medium chain triglycerides, beet root extract, and *Rhodiola rosea* root extract. One human equivalent dose (low dose) of 33 g was set at 1.1 g for rats weighing ~250 g. Major ingredients per 1 serving size or dose (human: 33 g, rat: 1.1 g) of the WPH-based supplement were then: 

Energy → human: 110 kcal, rat: 3.67 kcal;

Total fat → human: 1.5 g, rat: 0.05 g;

Total carbohydrate → human: 3 g, rat: 0.1 g;

Total protein → human 20 g, rat: 0.67 g;

Total leucine → human: 3.6 g, rat 0.12 g; and

Creatine → human: 2.5 g, rat 0.08 g.

The WPI powder (Mullins Whey Inc) used to compare the serum leucine and insulin responses in aim 1 was 92% protein dry weight basis and contained 2.58 g leucine per 33 g human serving (0.09 g per rat serving). Note that rat dosaging was performed per the methods of Reagan-Shaw et al. [12] whereby body surface area was taken into account in order to administer a human equivalent dose to rats for aim 1 as well as multiple doses for aim 2.

### **Circulating post-prandial insulin- and leucine-response profile of WPI versus the WPH-based supplement**

On the morning of testing, male Wistar rats (Charles Rivers Laboratories) aged 52–55 days (~250-300 g) had food removed at the beginning of the light cycle. Three hours later, each rat was gavage-fed a low dose (as above) of either WPI or the WPH-based supplement under light isoflurane anesthesia. The control condition (n = 4) was sacrificed without gavage-feeding in order to provide a baseline comparison point for fasting leucine and insulin values. Rats that were gavage-fed were subsequently sacrificed under CO_2_ gas at 15 (WPH n = 6, WPI n = 6), 30 (WPH n = 4, WPI n = 4), 60 (WPH n = 4, WPI n = 4) and 120 (WPH n = 4, WPI n = 4) minutes post gavage-feeding. A heart puncture using a 22-gauge needle was performed to collect whole blood into serum separator tubes and was subsequently centrifuged at 1300 rpm for 10 minutes in order to obtain serum. Of note, all of the aforementioned gavage-feedings took place between 1000–1600 hours.

Serum leucine concentrations were quantified using gas chromatography-electron impact-mass spectrometry (Agilent Technologies 6890 N capillary GC and 5973 Network Mass Selection Detector, Foster City, CA, U.S.A.) at the Washington University Biomedical Mass Spectrometry Research Resource. In brief, d_3_-leucine (10 nmol) was added as an internal standard to 100 μL serum. Serum amino acids were chemically converted to their trimethylsilyl form using N,O-Bis(trimethylsilyl)trifluoroacetamide + 10% Trimethychlorosilane (BSTFA + 10% TMCS, Regis, Morton Grove, IL), and selected ion intensities for mass/charge 158 (natural Leu) and 161 (d_3_-Leu) were monitored. Serum insulin was analyzed using an enzyme-linked immunosorbant assay specific for rat species according to manufacturer’s protocol (Millipore, Saint Charles, MO).

### **Toxicology assessment of chronic WPH supplementation**

The potential toxocologic effects of a low dose, medium dose, high dose of the WPH-based supplement as well as tap water only was examined over a 30-day period. The water only and low dose conditions required only one gavage feeding per day. The medium and high dose conditions required two and four gavage feedings per day, respectively, in order to: a) administer the required amount of protein to each rat, and b) to remain within the guidelines (1 ml/100 g) for stomach distension. Doses were recalculated per the aforementioned methods of Reagan-Shaw et al. [12] on a weekly basis during the 30-day feeding experiment in order to accommodate for rat growth from week to week.

Body composition using dual x-ray absorptiometry (DXA, Hologic QDR-1000/w) calibrated for small animals was performed on this cohort of animals after 7 days and 30 days of feeding in order to track alterations in body composition. Note that during this procedure, animals were placed under light isoflurane anesthesia so that the body scans could be performed.

Following the 30-day feeding schedule, animals were sacrificed under CO_2_ gas and blood and tissue samples were collected. Blood samples were obtained by cardiac puncture at sacrifice and the blood was collected in lithium heparin tubes. A complete blood count (CBC) was performed on whole blood using an automated hematology instrument (Hemavet 940FS, Drew Scientific, Dallas, TX). After completion of the CBC, the blood was centrifuged at 5,000 g for 5 minutes to separate the plasma. The plasma was harvested and a clinical biochemistry profile was performed on the plasma using an automated chemistry analyzer (AU640, Beckman-Coulter, Brea, CA) by Research Animal Diagnostics Laboratory (RADIL; Columbia, MO).

For tissue histology, a section of the left lateral and right medial liver lobes and both kidneys were collected, fixed overnight in 10% formalin and embedded in paraffin for histopathologic evaluation. Tissue sections were stained with hematoxylin/eosin and were examined for lesions by a veterinary pathologist specializing in rodent histopathology who was blinded to treatment status at RADIL. The body weight was recorded just after euthanasia and before bleeding, while heart and brain weights were measured after bleeding.

### **Statistics**

For aim 1, a two-way analysis of variance with Bonferroni corrections was performed to compare circulating leucine and insulin values in each postprandial rat condition to rats that were 3-h fasted. For aim 2, Chi-squared tests were performed in order to examine proportion differences in animals in each condition that presented signs of liver or kidney damage. One-way ANOVAs were performed for each serum/whole blood variable. For tracking changes in body composition variables, a two-way ANOVA (dose x time) was performed. Unless otherwise stated in figures and tables, all data were expressed as means ± standard error values and significance was set at p < 0.05.

## **Results**

### **Post prandial serum leucine and insulin differences between WPI and WPH**

Figureb 1A shows the leucine responses to the WPI and WPH-based supplement relative to rats that were not gavage-fed. In the WPI condition, serum leucine did not statistically increase relative to the control rats that were not gavage-fed. In contrast, WPH significantly increased at 15-min post-ingestion relative to the unfed control rats (p = 0.01). Importantly, a significant difference in circulating leucine at 15 minutes post-WPH gavage existed relative to 15 minutes post WPI-gavage (p = 0.04), but not at other time points.

**Figure 1:**
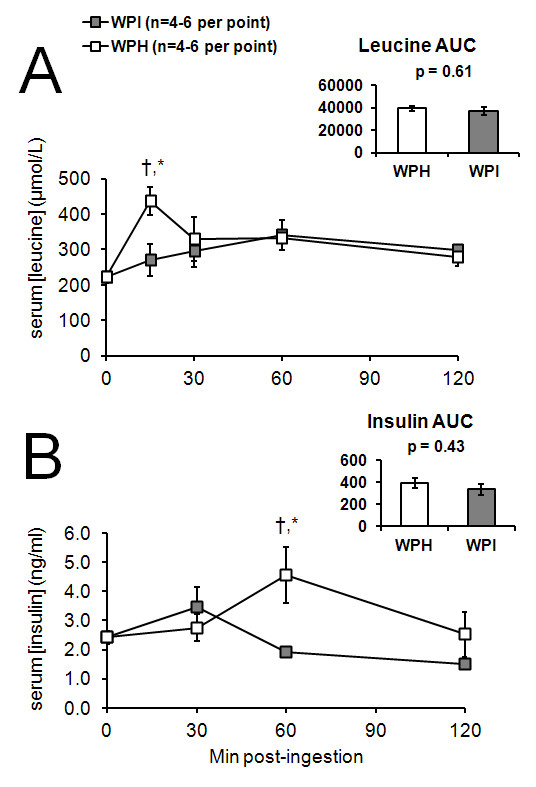
**Circulating postprandial leucine (A) and insulin (B) responses of a WPH-based supplement versus WPI.** Inset figures represent postprandial areas under the curve (AUCs) of each condition. All data are presented as mean ± SE; n = 4–6 rats per time point. Abbreviations/symbols: † = greater serum value than 3-h fasting concentrations for the respective supplement; * = WPH > WPI at a postprandial time point (p < 0.05).

Figureb 1B outlines the insulin responses to the WPI and WPH-based supplement. For post-WPI gavage, relative to the control rats that were not gavage-fed, no significant increases occurred in serum insulin at 60 minutes, and 120 minutes, although there tended to be an increase at 30 minutes post-gavage (p = 0.09). For post-WPH gavage, relative to the control rats that were not gavage-fed, a significant increase occurred in serum insulin 60 minutes post-WPH gavage (p = 0.01), while there were no significant increases in serum insulin at 30 minutes and 120 minutes (p > 0.05). Comparing the insulinogenic responses of both protein sources against one another at each time point importantly revealed that the WPH-based supplement elicited a significantly greater increase in insulin relative to WPI 60 minutes post-gavage (p = 0.001).

### **Body composition and food intakes following 30 days of feeding with different doses of the WPH-based supplement**

When comparing the low-dose WPH, medium-dose WPH, high-dose WPH, and water only, DXA analysis demonstrated that there were no significant between-condition differences from 7 days to 30 days in fat mass (dose x time interaction p = 0.90; Figureb 2). Similarly, there were no between-condition differences in total lean body mass (dose x time interaction p = 0.99) when comparing the low-dose WPH, medium-dose WPH, high-dose WPH, and water only (Figureb 2).

**Figure 2:**
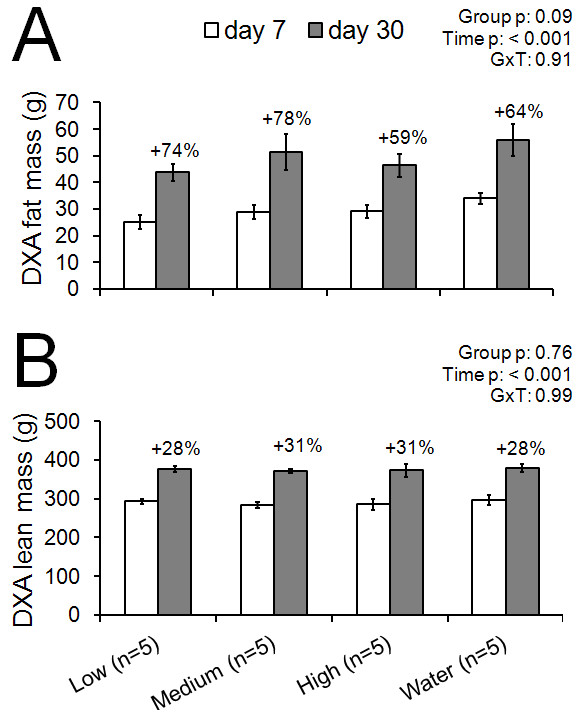
**Changes in DXA fat mass (A) and DXA lean mass (B) from days 7 to 30 of daily gavage feeding 1 human equivalent dose (1.1 g/d, ‘low’), 3 human equivalent doses (3.4 g/d, ‘medium’), and 6 human equivalent doses (6.8 g/d, ‘high’) of the WPH-based supplement as well as water only (‘water’).** All data are presented as mean ± SE and % changes from day 7 to day 30 are presented above each bar graph. No between-condition differences were detected.

As expected, progressive increases in the average amount of protein consumed per day were present from low to medium to high dosages (p < 0.05, Figureb 3A). Interestingly, there was also a significant difference between total energy consumed between WPH-based supplement conditions with the high dose exhibiting a significantly lower amount of food intake relative to the low-dose (p < 0.05, Figureb 3B) and water only condition (p < 0.01).

**Figure 3:**
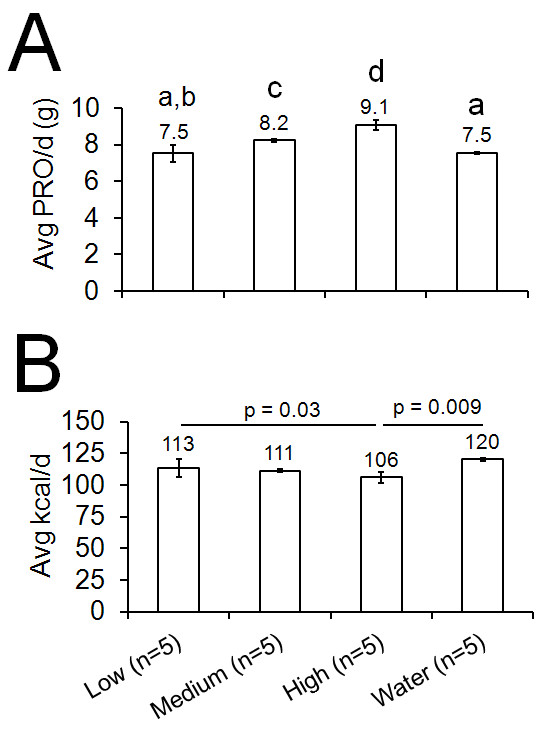
**Average daily protein (‘PRO/d’, A) and kilocalorie (‘kcal/d’, B) intake over the 30-day daily gavage feeding of 1 human equivalent dose (1.1 g/d, ‘low’), 3 human equivalent doses (3.4 g/d, ‘medium’), and 6 human equivalent doses (6.8 g/d, ‘high’) of the WPH-based supplement as well as water only (‘water’).** All data are presented as mean ± SE and daily averages are presented numerically above each bar. As expected, average protein intakes over the 30-day intervention (subfigure A) were as follows: high > medium > low = water (p < 0.01 denoted by different letters above each bar). Interestingly, energy intakes were significantly lower in the high condition relative to the low and water conditions (p-values presented above bars).

### **Liver and kidney histopathology and serum clinical chemistry profiles**

Histopathological assays conducted on the liver and kidneys after 30 days of low dose, medium dose or high dosages of the WPH-based supplement feeding showed no adverse effects on clinical pathology markers relative to water only feeding (Table 1). Interestingly, the proportion of rats fed water for 30 days (4/5 rats) presented significantly more >21 hepatocellular mitoses counts (representative of potential liver damage) relative to rats in the low (0%), medium (0%) and high WPH-based supplement conditions (0%, *X*^2^ p = 0.001).

**Table 1:** Dose-dependent effects of WPH feeding for 30 days on liver and kidney histopathology markers

Variable	water (n = 5)	low (n = 5)	medium (n = 5)	high (n = 5)
*Kidney histopathology*				
basophilia of tubules in corticomedullary junction	0/5	1/5	0/5	1/5
moderate unilateral hydronephrosis	0/5	0/5	0/5	1/5
large focal tubular regeneration with lymphocytes	0/5	0/5	1/5	0/5
focal tubular mineralization	0/5	1/5	0/5	0/5
focal perivascular lymphoid infiltrate	1/5	0/5	0/5	0/5
1-2 proteinaceous tubular casts	3/5	0/5	0/5	1/5
tubular regeneration with interstitial lymphocytes	1/5	0/5	0/5	1/5
focal tubular regeneration	2/5	0/5	2/5	1/5
*Liver histopathology*				
moderate multifocal centrilobular lipidosis	1/5	3/5	2/5	1/5
mild multifocal centrilobular lipidosis	3/5	2/5	2/5	3/5
few mild foci lipidosis	1/5	0/5	1/5	1/5
1-2 multinucleated giant cells	1/5	1/5	1/5	1/5
focal granulopoeisis	0/5	1/5	0/5	0/5
focal erythropoeisis	3/5	0/5	0/5	1/5
>21 hepatocellular mitoses	4/5^†^	0/5	0/5	0/5
11-20 hepatocellular mitoses	0/5	2/5	1/5	0/5
1-10 hepatocellular mitoses	1/5	3/5	4/5	1/5
6-10 apoptotic cells	3/5	0/5	1/5	0/5
1-5 apoptotic cells	2/5	5/5	3/5	3/5
6-10 microgranulomas	2/5	0/5	1/5	0/5
1-5 microgranulomas	3/5	2/5	3/5	3/5

Effects of 30 days of daily gavage feeding 1 human equivalent dose (1.1 g/d, ‘low’), 3 human equivalent doses (3.4 g/d, ‘medium’), and 6 human equivalent doses (6.8 g/d, ‘high’) of the WPH-based supplement as well as water only (‘water’) on markers of kidney and liver damage.

Liver apoptotic cell and microgranuloma counts represent potential liver injury/damage; hepatocellular mitoses and focal granuloposis/erythropoesis counts represent potential liver regeneration after injury; liver lipidosis counts represent the development of fatty liver.

Kidney histopathlogy definitions [derived from reference Guyton and Hall [13]:

Basophilia of tubules in corticomedullary junction counts represent potential nephron damage; moderate unilateral hydronephrosis represent excessive dilation of the kidneys and potential decrement in kidney function; large focal tubular regeneration with lymphocytes counts represent potential kidney damage and toxicity; focal tubular mineralization counts represent potential tubular damage; focal perivascular lymphoid infiltrate counts represent potential kidney damage and toxicity.

Liver histopathology definitions (derived from reference Guyton and Hall [13]):

Symbols: ^†^ indicates proportion of observations with water is significantly higher than observations in different treatment conditions as determined by a Chi-square test (p = 0.001).

There were no significant differences in serum clinical chemistry profiles between the 4 conditions (Table 2, p > 0.05). Finally, there were no significant differences in brain, heart, and whole body weights between the 4 conditions (Table 2, p > 0.05).

**Table 2:** Dose-dependent effects of WPH feeding for 30 days on blood and other health markers

Variable	p-value between conditions	water (n = 5)	low (n = 5)	medium (n = 5)	high (n = 5)
*Serum markers*					
Triglycerides (mg/dL)	p = 0.60	184 ± 28	169 ± 18	187 ± 13	153 ± 14
Glucose (mg/dL)	p = 0.32	183 ± 12	154 ± 11	187 ± 17	167 ± 14
Urea Nitrogen (mg/dL)	p = 0.45	25 ± 1	24 ± 1	26 ± 1	24 ± 2
Creatinine (mg/dL)	p = 0.25	0.41 ± 0.01	0.39 ± 0.01	0.44 ± 0.03	0.38 ± 0.02
Sodium (mmol/L)	p = 0.33	145 ± 1	147 ± 1	144 ± 1	146 ± 1
Potassium (mmol/L)	p = 0.20	6.4 ± 1	5.8 ± 0	6.9 ± 1	5.1 ± 0
Chloride (mmol/L)	p = 0.59	99 ± 0	98 ± 1	98 ± 0	99 ± 0
Total Protein (g/dL)	p = 0.17	6.9 ± 0.1	6.7 ± 0.1	6.7 ± 0.1	6.5 ± 0.0
Albumin (g/dL)	p = 0.26	3.5 ± 0.0	3.4 ± 0.0	3.4 ± 0.1	3.4 ± 0.1
Calcium (mg/dL)	p = 0.06	12.8 ± 0.1	12.5 ± 0.2	12.4 ± 0.3	12.0 ± 0.1
Phosphorus (mg/dL)	p = 0.40	10.8 ± 0.5	10.6 ± 0.6	11.7 ± 0.5	10.5 ± 0.4
Cholesterol (mg/dL)	p = 0.34	82 ± 10	64 ± 3	68 ± 7	74 ± 7
Total Bilirubin (mg/dL)	p = 0.08	0.10 ± 0.0	0.10 ± 0.0	0.14 ± 0.0	0.10 ± 0.0
ALT (U/L)	p = 0.68	239 ± 43	254 ± 54	298 ± 34	234 ± 27
ALP (U/L)	p = 0.52	186 ± 16	179 ± 11	161 ± 4	165 ± 18
GGT (U/L)	p = N/A	<3	<3	<3	<3
Total CO_2_ (mmol/L)	p = 0.14	33 ± 1	37 ± 2	32 ± 2	33 ± 1
*Whole blood markers*					
WBC (x10³/μL)	p = 0.88	12.5 ± 0.9	11.3 ± 1.2	12.0 ± 1.2	11.8 ± 0.5
Seg. Neutro (x10³/μL)	p = 0.85	1.7 ± 0.2	1.7 ± 0.6	1.3 ± 0.3	1.8 ± 0.3
Band Neutro (x10³/μL)	p = 0.99	0.0 ± 0.0	0.0 ± 0.0	0.0 ± 0.0	0.0 ± 0.0
Lymphocytes (x10³/μL)	p = 0.74	10.7 ± 0.9	9.6 ± 0.7	10.5 ± 1.0	9.8 ± 0.5
Monocytes (x10³/μL)	p = 0.32	0.07 ± 0.03	0.00 ± 0.00	0.06 ± 0.04	0.05 ± 0.03
Eosinophils (x10³/μL)	p = 0.92	0.12 ± 0.09	0.09 ± 0.07	0.09 ± 0.05	0.16 ± 0.10
Basophils (x10³/μL)	p = 0.99	0.0 ± 0.0	0.0 ± 0.0	0.0 ± 0.0	0.0 ± 0.0
RBC (M/μL)	p = 0.47	8.5 ± 0.1	8.4 ± 0.1	8.6 ± 0.2	8.7 ± 0.1
Hemoglobin (g/dL)	p = 0.08	16.1 ± 0.3	16.9 ± 0.3	16.3 ± 0.2	16.8 ± 0.2
Hematocrit (%)	p = 0.75	52.7 ± 1.1	53.4 ± 0.9	52.7 ± 1.1	53.8 ± 0.5
MCV (fL)	p = 0.29	61.7 ± 0.8	63.5 ± 0.7	61.5 ± 0.9	61.8 ± 0.7
MCH (pg)	p = 0.01	18.8 ± 0.3^a^	20.1 ± 0.2^b^	19.1 ± 0.3^a^	19.3 ± 0.2^c^
MCHC (g/dL)	p = 0.08	30.5 ± 0.3	31.7 ± 0.2	31.1 ± 0.5	31.2 ± 0.1
Cell Volume (%)	p = 0.19	49.8 ± 0.9	51.4 ± 0.4	49.8 ± 0.6	50.6 ± 0.2
Platelets (x10³/μL)	p = N/A	Clumps	Clumps	Clumps	Clumps
Hemolysis	p = N/A	Clear	Clear	Clear	Clear
MPV (fL)	p = 0.38	6.7 ± 0.1	6.3 ± 0.2	6.7 ± 0.3	6.5 ± 0.2
*Post necropsy organ and body weights*					
Brain (g)	p = 0.57	2.03 ± 0.03	2.08 ± 0.04	2.08 ± 0.02	2.04 ± 0.06
Heart (g)	p = 0.88	1.40 ± 0.07	1.37 ± 0.04	1.35 ± 0.04	1.40 ± 0.05
Whole Body (g)	p = 0.69	439 ± 14	422 ± 9	419 ± 2	422 ± 20

Effects of 30 days of daily gavage feeding 1 human equivalent dose (1.1 g/d, ‘low’), 3 human equivalent doses (3.4 g/d, ‘medium’), and 6 human equivalent doses (6.8 g/d, ‘high’) of the WPH-based supplement as well as water only (‘water’) on clinical chemistry serum and whole blood markers.

Abbreviations (definitions): *ALT* = alanine aminotransferase (liver enzyme); *ALP* = alkaline phosphatase (liver and bone enzyme); *GGT* = gamma-glutamyl transpeptidase (liver enzyme); *WBC* = white blood cells; Seg. *Neutro*. = segmented neutrophils; *RBC* = red blood cells; *MCV* = mean corpuscular volume (measure of RBC size); *MCH* = mean corpuscular hemoglobin (average hemoglobin per red blood cell); *MCHC* = mean corpuscular hemoglobin concentration (average hemoglobin concentration per red blood cell); *MPV* = mean platelet volume (average size of platelets in whole blood).

Note that the ‘low’ condition presented significantly greater MCH content relative to the water and medium conditions (denoted by letter superscripts, p < 0.05).

## **Discussion**

Our data suggest that the tested WPH-based supplement: a) exerted a transient rise in post-prandial leucine and a subsequent insulin rise relative to WPI during a post-absorptive (not fasted) state at the low dose; and b) did not adversely affect markers of kidney, liver, and or other health markers after a 30-day feeding period, while decreasing food intakes in a dose-dependent fashion. Together, these data regarding serum responses to the tested WPH-based supplement can be considered to be a promising lead for future experiments, which would aim to continue examining the physiological effects that WPH-based protein sources exhibit on other tissues such as skeletal muscle and adipose tissue.

It has been shown that extracellular leucine availability, with or without exercise, increases muscle protein synthesis rates [3,14-17]. Likewise, the insulinogenic effects of whey have been posited to potentially aid in augmenting muscle protein synthesis in an mTORC1-dependent fashion independent of intramuscular mRNA expression patterns [18], although this effect has been suggested to be more permissive rather than stimulatory [14]. In agreement with previous evidence, our data demonstrates that WPH has been shown to be insulinogenic at one hour following feeding in humans [3], albeit their data was collected after an overnight fast. The mechanism whereby whey elicits its superior insulinogenic effects relative to other protein sources may be related to unidentified bioactive peptides and/or its amino acid profile; specifically arginine [19]. However, both protein sources in our study possessed nearly similar amounts of arginine (WPH-based supplement: 470 mg per human serving, WPI = 510 mg). Nonetheless, our data suggests that WPH may be superiorly insulinogenic relative to an undigested whey protein source; an effect which we speculate could be due either: a) its superior effect in stimulating the transient increase in postprandial serum leucine given that leucine has been shown to stimulate insulin secretion [20], or b) the presence of unidentified bioactive peptides that occur due to the hydrolysis process which stimulate pancreatic insulin secretion. In regards to the later, Morifuji et al. [21] have determined that dipeptides from WPH stimulate muscle glucose uptake via PI3-kinase and protein kinase C (PKC) pathways. Therefore, existing evidence in the literature, demonstrates that WPH-based peptides exhibit significant physiological effects on the pancreas warrants future research into elucidating mechanisms that drive these phenomena.

As mentioned previously, WPH has been shown to elicit a transient leucine spike in the serum, although this effect has only been shown under fasting conditions and when comparing WPH to casein and soy [3]; of note WPI and WPH have been examined for branched chain amino acid responses, but not leucine responses explicitly [7]. Fasting rats for 12 hours prior to feeding them a high-protein test meal yielded serum leucine concentrations that were 60% lower than the rats in our study after 3 hours of food removal [22] which implies that our animals were in a post-absorptive (not fasted) state. However, we chose to examine the leucine responses between the WPH-based versus WPI after a 3-h food withdrawal with the notion that humans would likely consume the whey protein-based supplement prior to or following an exercise bout within 3–6 hours of consuming a meal, as most humans eat throughout the wake cycle. Therefore, this is the first report to our knowledge demonstrating that subjects in the post-absorptive state exhibit greater leucine and subsequent insulin responses when ingesting a hydrolyzed whey protein source versus a native whey protein isolate.

We also report that 30 days of chronic supplementation with a WPH-based supplement in rodents aged 62 days old when study began: a) causes no apparent adverse health effects on the kidneys and/or liver, b) does not affect brain and/or heart weights, c) does not affect circulating clinical chemistry and whole blood markers, and d) does not alter body composition. As mentioned previously, studies in healthy humans have demonstrated that higher protein intakes seemingly exert no adverse effects on markers of renal or liver function [9,10]. Resistance training studies have also determined that increasing protein intakes for two months did not negatively impact serum clinical chemistry markers related to kidney and liver damage [23,24]. However, concern still exists in the medical literature regarding the potential negative effects that protein supplementation exerts on liver [11,25] and kidney physiology [25,26]. While limited data exists on the safety of chronic whey protein supplementation, little data to our knowledge has utilized a rodent model whereby liver and kidney tissues were morphologically examined for lesions following chronic feeding. One recent study [27] did determine that 18 days of WPI consumption offset liver toxicity caused by the concomitant administration of a pro-oxidant agent (dimethylnitrosamine). Interestingly, we determined that only the water condition presented a greater incidence of liver damage (> 21 hepatocellular mitoses) relative to the WPH-supplemented conditions. We speculate that WPH or whey protein supplementation in general supplementation could indeed be hepatoprotective. Of note, the WPH supplement contained *Rhodiola rosea* extract which is a well-known adaptogen that confers hepatoprotective (i.e., antioxidant and antilipidemic) effects in db/db mice [28]. Whether it is the WPH fraction and/or the *Rhodiola rosea* extract in the WPH-based supplement, we conclude that the WPH-based supplement used in our study does not exacerbate liver damage when administered in very high doses and could, instead, confer hepatoprotective effects.

Contrary to the one referenced study examining the effects of whey protein on liver histopathology markers in rodents [27], our study is seemingly the first to suggest that 30 days of feeding a range of WPH-based protein dosages to rats does not negatively impact kidney damage/toxicology markers and/or circulating markers of kidney function (i.e., creatinine and blood urea nitrogen). Rats in the high dose condition consuming 6 human equivalent doses per day (would be equivalent to an additional 120 g of protein in humans) increased daily protein intakes up to 21.7 g/kg/day. Additionally, 30-days of creatine feeding present within the WPH-based supplement did not adversely affect the examined health markers; for the high dose condition this would be equivalent to a human consuming 15 g/d of creatine. Therefore, our 30-day study is in agreement with other literature which continues to refute speculation that whey protein [9,10] and/or creatine supplementation [29] negatively impacts kidney function and/or elicits kidney damage in animals that do not possess pre-existing kidney issues.

Interestingly, animals that were gavage-fed three and six human equivalent doses per day of the WPH-based supplement for 30 days consumed less total kilocalories per day relative to animals that consumed one human-equivalent dose and water over this time frame. Multiple studies have established that whey protein may exert satiating effects and reduce adiposity in rats [30,31]. In explaining this effect, authors from the later study propose that whey-derived proteins do elicit a satiating effect through the enhanced secretion of gut neuropeptides including cholecystokinin (CCK) or glucagon-like peptide-1 (GLP-1). Thus, this effect might have been observed in our study although examining circulating CCK and GLP-1 was beyond the scope of our investigation. With regard to body composition alterations, however, the feeding intervention in our study did not confer changes in body fat in the protein supplemented conditions. Likewise, the feeding intervention did not increase DXA lean body mass which has been demonstrated in the aforementioned rodent study that chronically fed rats whey protein over a 25-day period [31]. However, that Pichon et al. [31] used dissection methods to assess body composition whereas our DEXA method may introduce a larger degree of error which could have obscured our findings. Furthermore, we cannot rule out the hypothesis that consuming higher protein diets over longer periods (i.e., years to decades in humans) reduces adiposity and enhances and/or maintains muscle mass during maturation and subsequent aging in humans, respectively.

It is also noteworthy mentioning that there are limitations to the current study. First, rodents were examined instead of humans with regards to studying leucine, insulin, and toxicological responses to these whey protein sources. It should be noted, however, that rats and humans seem to respond similarly to whey protein as it has been shown to increase circulating leucine and markers of muscle protein synthesis following exercise in both species [3,32]. Thus, we hypothesize that human responses will likely be similar when examining the physiological effects of WPH versus WPI supplements. With regard to the current toxicology study, it should be noted that only 5 animals were examined per condition over a 30-day feeding period. In parallel to our study, however, there are other recent studies examining the toxicological effects of other compounds which have similarly studied 6 animals per condition [33,34]. Creatine monohydrate (equivalent to 2.5 g/dose for humans) is also a major ingredient in the WPH-based supplement. However, creatine monohydrate does not alter glucose tolerance or insulin sensitivity and is not insulinogenic nor does it affect circulating leucine concentrations [35]. With regard to other major ingredients present in the WPH-based supplement, L-citrulline has not been shown to impact circulating insulin and/or leucine levels [36], although vitamin C has been shown to reduce insulin in type II diabetes patients over chronic supplementation periods [37], and L-lysine may stimulate insulin secretion from pancreatic beta cells [38]. Therefore, beyond the active biopeptides that exist in the WPH formulation, other ingredients may have influenced the insulin response. Finally, while we examined the postprandial circulating leucine response to a WPH-based supplement versus WPI, it remains unknown as to whether or not potential unknown biologically active peptide fragments that occur during the whey hydrolysis process spike in the bloodstream after feeding relative to WPI [this aspect of food science is reviewed in [39]. In this regard, future animal and/or human studies should pursue this exciting and unexplored nutraceutical research area in order to determine if WPH supplementation with exercise confer positive skeletal muscle anabolic responses due to potential increases in circulating bioactive peptide fragments relative to other protein sources.

## **Conclusions**

In summary, our rodent feeding model uniquely found that the WPH-based supplement elicited greater transient leucine with a subsequent increased insulin response relative to the WPI. Given these data in conjunction with the recent data demonstrating that WPH may possess biologically active peptide fragments [5], it will be of future interest to compare the anabolic effects of WPI- versus WPH-based supplements surrounding resistance training and/or the effect of WPH-based supplements in persons with diminished insulin secretion. Our 30-day feeding rodent model suggests that WPH-based supplements are safe to consume for one month in rats and may confer satiating effects which reduced total food intake, albeit the relatively short-term feeding study did not unveil significant alterations in total fat mass between the administered dosages. In this regard, longer-term human studies might be performed in order to examine the potential weight regulatory effects that WPH-based products (i.e., meal-replacement shakes) may exhibit on overweight and obese populations.

## **Competing interest**

The authors declare no competing interests.

## **Authors’ contributions**

RGT assisted with: 1) data collection; 2) data analysis; 3) statistical analysis; 4) preparing manuscript. TEC assisted with: 1) intellectual contribution throughout experiments; 2) manuscript preparation. SRH Performed histological examination of kidney and liver tissues and provided intellectual contribution throughout experiments. JRC performed leucine analysis. FWB assisted in: 1) study design; 2) intellectual contribution throughout experiments; 3) manuscript preparation. MDR procured grant funding; assisted in: 1) study design; 2) data collection and analysis; 3) preparing manuscript. All authors read and approved the final manuscript.
